# The *Lingulodinium* circadian system lacks rhythmic changes in transcript abundance

**DOI:** 10.1186/s12915-014-0107-z

**Published:** 2014-12-20

**Authors:** Sougata Roy, Mathieu Beauchemin, Steve Dagenais-Bellefeuille, Louis Letourneau, Mario Cappadocia, David Morse

**Affiliations:** Département de Sciences Biologiques, Université de Montréal, Institut de Recherche en Biologie Végétale, 4101 Sherbrooke est, Montréal, Québec H1X 2B2 Canada; Centre d’Innovation Génome Québec, McGill University, 740 Docteur Penfield, Montréal, Québec H3A 1A4 Canada

**Keywords:** Transcript profiling, RNA Seq, Circadian rhythms, Dinoflagellate

## Abstract

**Background:**

Almost all cells display circadian rhythms, approximately 24-hour period changes in their biochemistry, physiology or behavior. These rhythms are orchestrated by an endogenous circadian clock whose mechanism is based on transcription-translation feedback loops (TTFL) where the translated products of clock genes act to inhibit their own transcription.

**Results:**

We have used RNA-Seq to measure the abundance of all transcripts in an RNA-Seq-derived *de novo* gene catalog in two different experiments. One compared midday and midnight in a light–dark cycle (ZT6 and ZT18) and under constant light (CT6 and CT18). The second compared four different times (ZT2, ZT6, ZT14 and ZT18) under a light dark cycle. We show here that despite an elaborate repertoire of biological rhythms, the unicellular dinoflagellate *Lingulodinium* had no detectable daily variation in the abundance of any transcript in an RNA-Seq-derived *de novo* gene catalog. We also examined the timing of the bioluminescence and photosynthesis rhythms in the presence of the transcription inhibitors actinomycin D and cordycepin. We found that the timing of the two rhythms was unchanged even when transcription rates had decreased to roughly 5% the levels of untreated cells.

**Conclusions:**

The lack of detectable daily variation in transcript levels indicates that the endogenous circadian timer of *Lingulodinium* does not require rhythmic RNA. If the circadian timer is considered as a limit cycle oscillator, then cellular time in this organism must be defined by variations in state variables that do not include the amount of a clock gene transcript.

**Electronic supplementary material:**

The online version of this article (doi:10.1186/s12915-014-0107-z) contains supplementary material, which is available to authorized users.

## Background

Circadian rhythms are changes in a cell’s biochemistry, physiology or behavior that occur with a roughly 24-hour period. The rhythms are the observable outputs of a cell-autonomous and resettable timekeeper [1] called a circadian clock. This endogenous circadian clock is ubiquitous and organized as transcription-translation feedback loops (TTFL) in eukaryotes, and although the genes that constitute the clock differ between plant, animal and fungal model systems [2], transcriptional control is thought to be an integral component of the clock mechanism. Even in cyanobacteria, where a daily rhythm in the phosphorylation state of the clock protein KaiC has been proposed to be the pacemaker [3], a KaiC TTFL may be required for generating robust rhythms [4].

Recent observations, however, have indicated that transcription is not always required to produce circadian oscillations, as evidenced by the daily oscillation of redox cycles of peroxiredoxin in red blood cells [5] and the green algae *Ostreococcus tauri* [6]. Interestingly, when *O. tauri* is placed in prolonged darkness, transcription rates fall below detectable limits and the rhythm in luciferase fused with the clock component CCA (Circadian Clock associated) dampens after one day. However, when rhythmicity is re-initiated by transfer to constant light, the phase of the rhythm varies depending on the time when cells are exposed to light [6]. Thus, either a timer driving the observed rhythm of this translational reporter continues even though the overt rhythm itself is undetectable, or the TTFL of *O. tauri* may be influenced by cross-talk with the non-transcriptional peroxiredoxin rhythm which has been shown to continue unabated in darkness.

The marine dinoflagellate *Lingulodinium* displays a large variety of overt rhythms and has been a model for study of the mechanisms linking the clock with these rhythms for many years [7]. For example, the bioluminescence rhythm is correlated with rhythmic changes in the amount of the reaction catalyst (dinoflagellate luciferase) [8] and of a luciferin binding protein (LBP) [9] that protects the bioluminescence substrate luciferin from non-bioluminescent oxidation. In addition, the sequestration of the key carbon-fixing enzyme ribulose-1,5-bisphosphate carboxylase/oxygenase (Rubisco) within the pyrenoid of the chloroplast is correlated with the capacity of the cell to fix carbon efficiently [10]. Both these different rhythms correlate with rhythms in the rate of protein synthesis *in vivo*, indicating that clock control over gene expression may regulate the timing of the rhythms. Importantly, in these and other examples, the control over protein synthesis occurs at a translational level since levels of the corresponding mRNAs do not change over the daily cycle [9,11-13].

In contrast to the depth of knowledge concerning the rhythms, the mechanism of the circadian clock in *Lingulodinium* remains unknown. To compound the difficulty in characterizing the central timer, physiological studies have shown that these single-celled organisms actually contain two different endogenous clocks, as the rhythms of bioluminescence and swimming behavior can run with different periods [14] and show different phase resetting behavior [15]. In the present study, we looked for rhythmic transcripts in *Lingulodinium* in order to identify potential TTFL components. We used RNA-Seq to assess levels of all RNA species in a *Lingulodiunium* transcriptome [16] over both diurnal and circadian cycles. Surprisingly, our analyses indicate that *Lingulodinium* does not express any detectable rhythmic transcripts. This suggests that the mechanism of the endogenous timers in this organism will instead involve translational and post-translational mechanisms.

## Results

**Figure 1:**
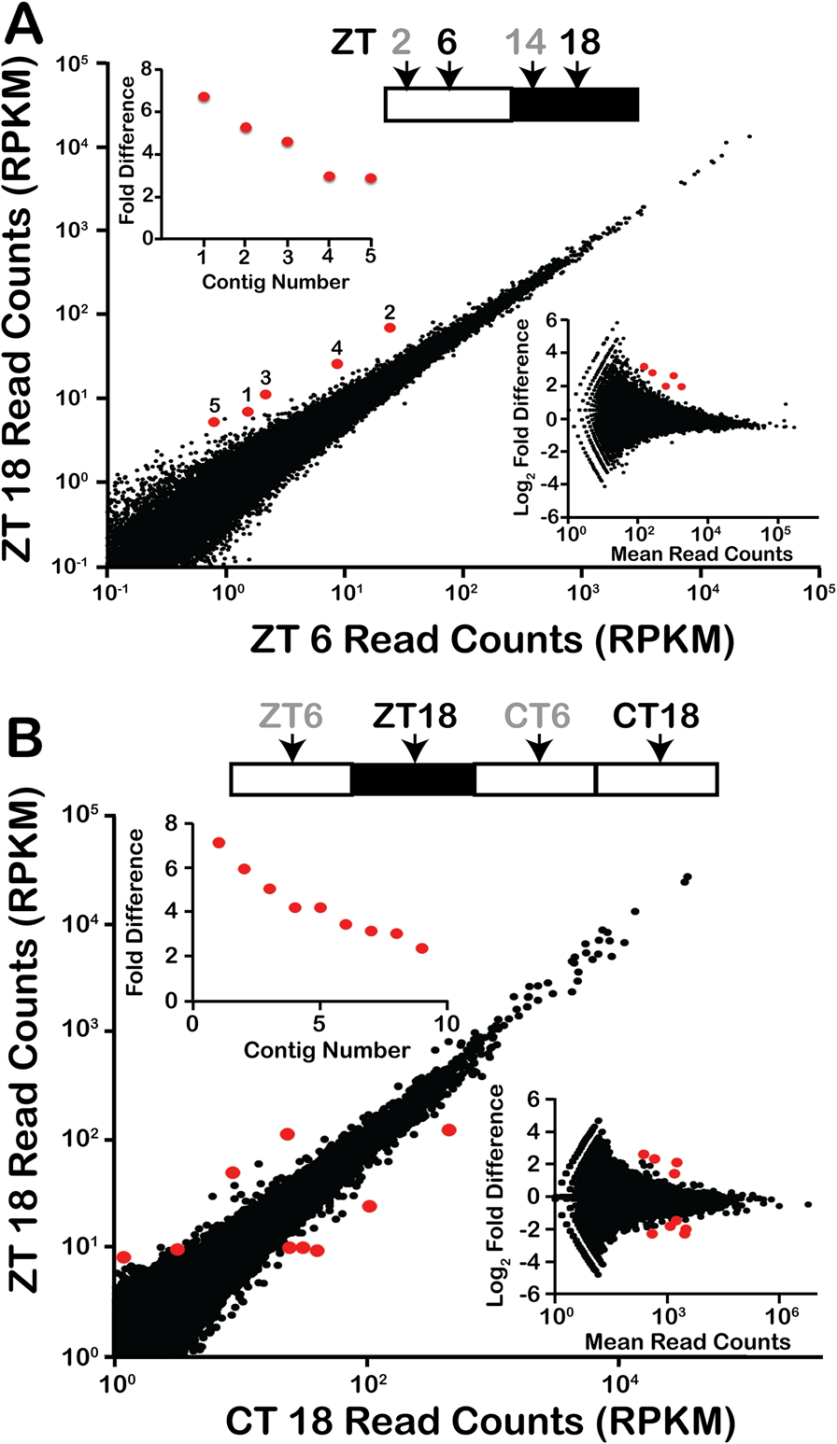
**Transcript abundance does not change between midday (ZT 6) and midnight (ZT 18). (A)** Read counts from the two samples (as reads per kilobase per million, RPKM), obtained by mapping raw read data to 103,266 contig Trinity assembly, were plotted against each other. Contigs corresponding to rRNA were removed. Insets show (upper left) the fold difference for DESeq statistically significant differences (*P*_adj_ <0.001) and (lower right) the MA plot used to determine statistically valid differences. **(B)** Transcript abundance does not change between true (ZT 18) and subjective (CT 18) midnight. CT, circadian time; MA, Magnitude-Average; ZT, Zeitgeber time.

To assess the possibility of isolating components of a transcription-based oscillator in a dinoflagellate, RNA-Seq was used to quantitate transcript levels globally at different times. Two different RNA-Seq experiments were performed, the first of which generated 252 million 76 bp paired end reads (using Zeitgeber times ZT 6 and ZT 18, and circadian times CT 6 and CT 18) while the second generated 545 million 100 bp paired end reads (taken at times ZT 2, ZT 6, ZT 14 and ZT 18), of which 51% and 92%, respectively, mapped to our *de novo* gene assemblies. We first compared ZT 6 and ZT 18 by mapping the 100 bp reads to a 103,266 contig Trinity assembly [17] (Figure 1A), expecting to find both light-induced and circadian differences between the two times. Instead, read counts from the two times, normalized as reads per kilobase per million reads (RPKM) [18] show surprisingly few differences in mRNA levels. DESeq [19] analysis indicated that only five contigs showed significantly different levels between the two times (*P*_adj_ <0.001). A similar result was obtained when the reads were mapped to a 74,655 contig Velvet assembly [16] for which DESeq identified 13 significant differences (*P*_adj_ <0.001) (see Additional file 1: Figure S1A). Of the five significant differences uncovered using the Trinity assembly, three were also identified as significant using the Velvet assembly, while the remaining two do not have corresponding contigs in the Velvet assembly. The increased number of significant differences in the Velvet assembly is primarily due to slight differences in the read counts that move either the fold difference or the mean read counts above the significance threshold. Similar results were obtained using EdgeR [20] to assess significant differences, so DESeq was used for all further analyses.

To confirm the similarity in transcript levels between midday and midnight, the midday/midnight comparison was repeated using the 76 bp paired-end read experiment. This experiment included samples taken at both true (ZT 18) and at subjective (CT 18) midnight, so we first assessed the similarity between these two. DESeq analysis identified nine and six significant differences when read counts were mapped to the Trinity (Figure 1B) and Velvet (see Additional file 1: Figure S1B) assemblies, respectively, with only one contig common to both. We conclude from this that constant light during the night phase does not cause a major alteration in gene expression, and, thus, the two midnight samples are essentially duplicates. We, therefore, compared the duplicate midday samples with these two midnight samples using DESeq (see Additional file 2: Figure S2). This analysis revealed no significant differences using the Trinity assembly and only three significant differences with the Velvet assembly. Since none of the differences found in this second experiment were in common with those found in the first experiment, we conclude that the mRNA complement of the cells at midday is the same as at midnight.

**Figure 2:**
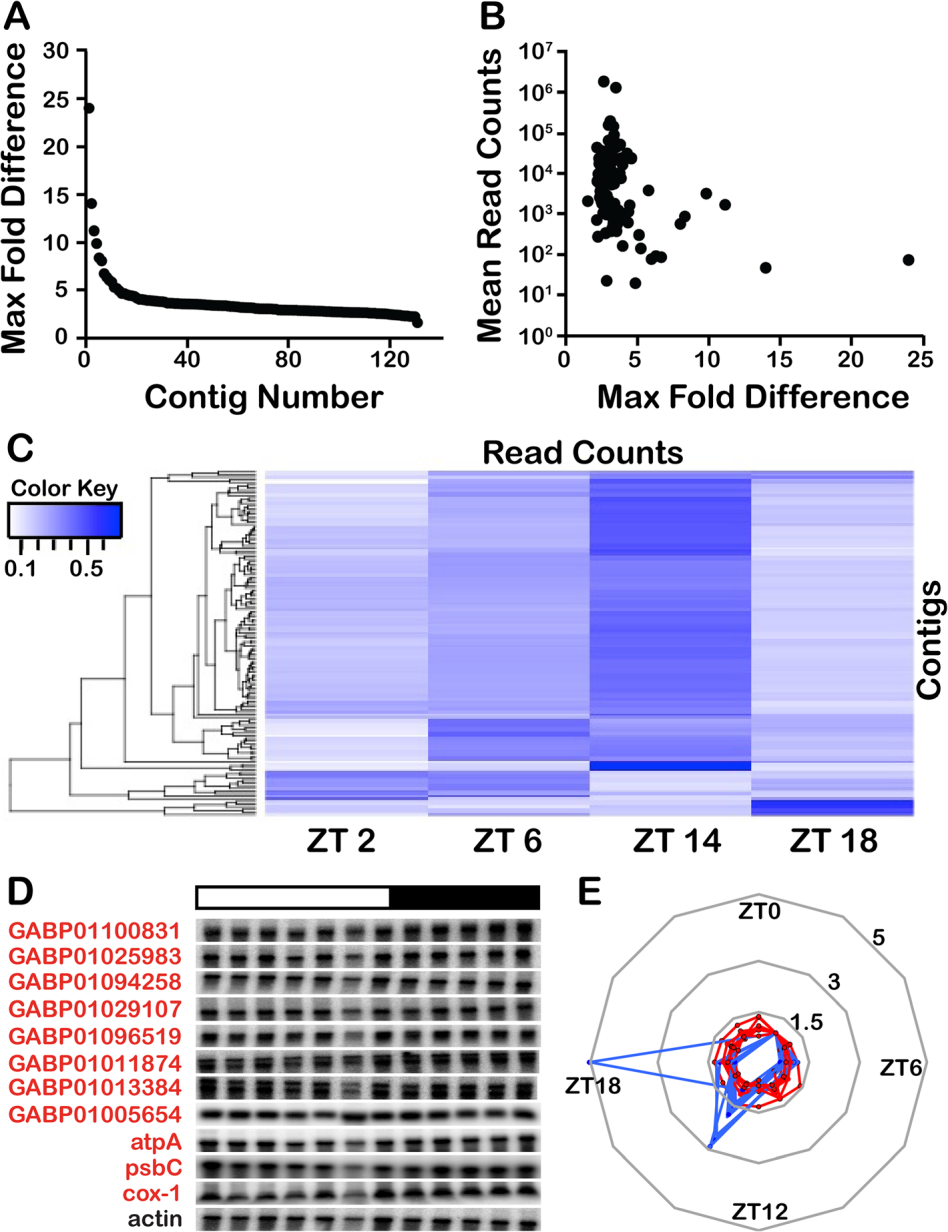
***Lingulodinium*** **does not have rhythm transcripts over a 24-hour cycle. (A)** DESeq significant differences for all pairs of samples taken LD 2, 6, 14 and 18 under a light dark cycle were combined to a single list of 131 contigs. **(B)** Contigs with more than five-fold maximum difference are typically those with lower mean read counts. **(C)** A heat map of all the significant differences detected shows a preponderance of contigs whose abundance appears greatest at ZT 14. Hierarchical clustering was performed only with rows to preserve the order of the time points. **(D)** Northern blots using probes prepared from a random selection of contigs indicate the RNA-Seq predicted differences are false positives. **(E)** Densitometric scans of Northerns relative to actin as a loading control (red) compared to normalized RNA-Seq data (blue). LD, light/dark; ZT, Zeitgeber time.

To test the possibility that cells might express rhythmic RNA with maxima lying between midday and midnight, we next compared the two additional times (ZT 2 and ZT 14) in the 100 bp read experiment. These times, immediately after the light/dark transitions, were chosen to maximize the chances of finding differentially accumulated RNAs. We determined significant differences from all possible pairwise comparisons of read counts mapped to the Trinity assembly using DESeq (*P*_adj_ <0.001) and combined all to yield a final list of 131 non-rRNA sequences (Figure 2A). Those showing the greatest fold-differences were typically lower in abundance (Figure 2B). Hierarchical clustering identified four main groups of sequences, with those peaking at ZT 14 containing the greatest number of significantly different RNAs (Figure 2C). Surprisingly, however, among the 42 sequences that could be identified by sequence homology were found two of the three known mitochondrial protein-coding genes [21] and nine of the ten known plastid protein-coding genes [22], and all these organelle-encoded transcripts were among the group with peak expression at ZT 14. The plastid-encoded sequences included the *psbA* transcript that had been previously shown by Northern analyses to be arrhythmic [23], suggesting that at least some of the transcripts identified as significantly different in our high throughput approach might in fact be false positives. To assess this possibility, Northern analyses were performed using a random selection of 11 sequences (Figure 2D). No rhythmicity was observed in the levels of any of these RNAs, as confirmed by densitometric scans (Figure 2E). Taken together, the two RNA-Seq experiments thus indicate that the entire mRNA complement of the cell is maintained at constant levels over the 24-hour cycle, both under light/dark cycles or in constant light.

**Figure 3:**
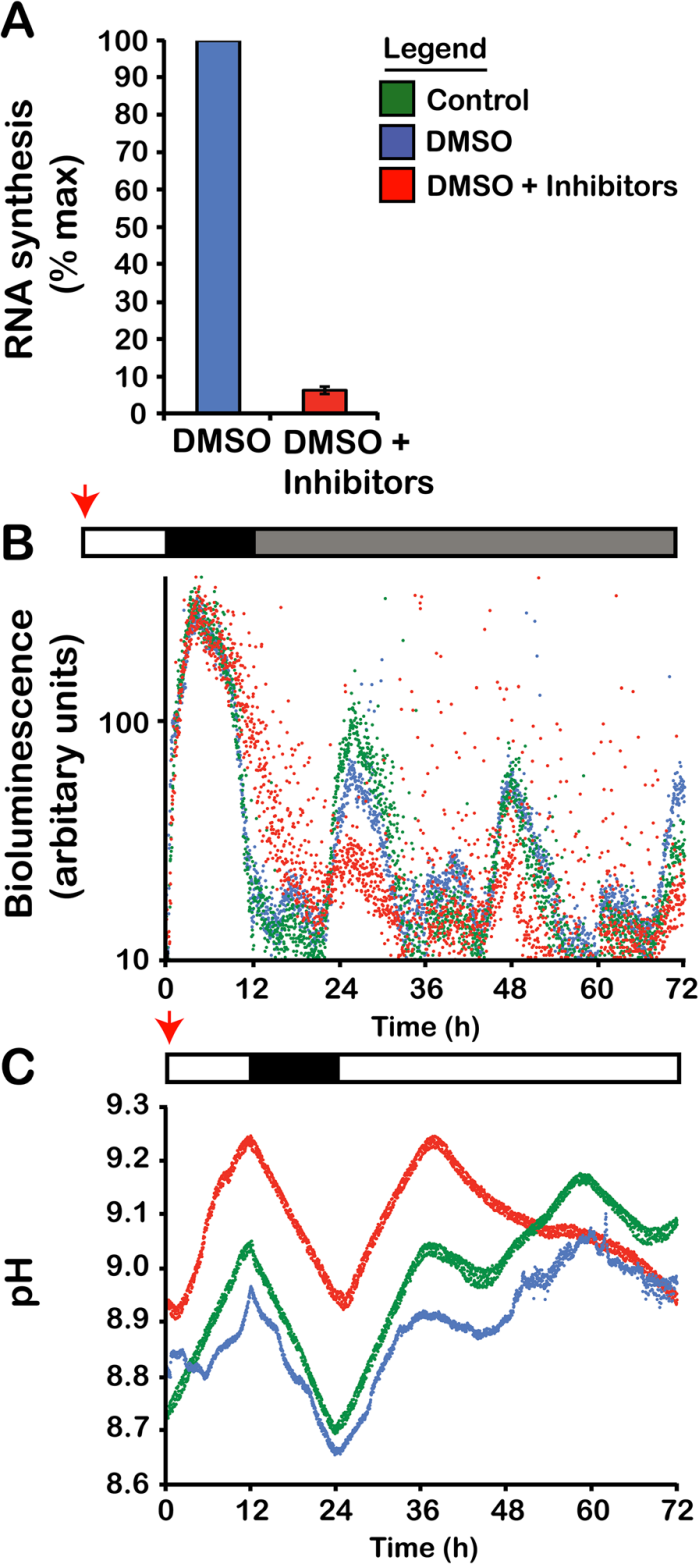
**Rhythms continue in the presence of transcription inhibitors. (A)** A combination of actinomycin D and cordycepin reduces RNA synthesis rates to around 5% (mean ± SD, n = 4) of the values of vehicle treated cultures. Cultures survive three to five days at these concentrations of inhibitors. **(B)** There is no change in the timing of the bioluminescence rhythm in constant dark. Horizontal bars indicate the light regime, with drugs added to the cultures at the indicated times (red arrows). Each data point is the average of three samples. **(C)** There is no change in the timing of the pH rhythm used to monitor CO_2_ fixation. A representative example of three different experiments is shown for each treatment. The amplitude of the rhythm is severely diminished by the third day of treatment with inhibitors. SD, standard deviation.

The absence of rhythmic RNAs suggested that the circadian clock would continue unabated in the presence of transcription inhibitors. To test this, we exposed cell cultures to a combination of the transcription inhibitors actinomycin D and cordycepin (3-doxyadenosine). Actinomycin D binds to DNA and interferes with transcription and replication [24] while cordycepin interferes with polyadenylation of mRNAs [25]. This combined treatment reduced RNA synthesis to levels roughly 5% of vehicle-treated cultures (Figure 3A) and is lethal after three days. Importantly, within this three-day time frame, the presence of the inhibitors does not change the timing of the bioluminescence rhythm in constant darkness (Figure 3B) although the amplitude of the rhythm shows a marked decrease. Furthermore, the timing of the photosynthesis rhythm in constant light (Figure 3C) appears similar in the presence of the drug, although again we note a marked decrease in the rhythmic amplitude, especially during the third day (the second day under constant conditions). We conclude from these experiments that, despite a strong effect on the amplitude of the two rhythms, robust transcription is not required for determining their circadian timing.

## Discussion

The results described here indicate that none of the sequences in our *Lingulodinium* transcriptomes show circadian or diurnal variations in transcript levels (Figures 1, 2). These analyses rely on assemblies, derived from high throughput Illumina RNA-Seq experiments that have been constructed using either Velvet or Trinity. The Velvet assembly has been most extensively characterized, with the 74,655 contigs estimated to represent approximately 94% of the total transcripts expressed by the cells [16]. The 103,266 contig Trinity transcriptome contains all the Velvet contigs as well as roughly 28,000 additional contigs [17]. Thus, it seems unlikely that *Lingulodinium* will express a rhythmic transcript in an RNA species that is not represented in either of the two assemblies. This conclusion is also supported by the finding that reducing the transcription rate to 5% of control levels does not affect the period of either the bioluminescence or the photosynthesis rhythms (Figure 3).

The manifestation of eukaryotic circadian rhythms in the absence of transcription was first suggested by observations of the photosynthesis rhythm in enucleated *Acetabularia* [26]. More recently, the finding of a circadian rhythm in peroxiredoxin redox state in red blood cells [5], the demonstration of a FRQ-less oscillator in *Neurospora* [27] and the ability of transcription-incompetent *O. tauri* [6] to keep time in constant darkness have also provided support for this view. However, since these latter two organisms also have a canonical TTFL, the significance of a non-transcriptional oscillator operating in parallel remains unclear. In contrast, our findings indicate that the entire *Lingulodinium* circadian system has evolved to function without a TTFL, a particularly remarkable finding considering that there are at least two independent circadian timers in these cells [14]. It is unknown at this time if *Lingulodinium* will also show a circadian rhythm in peroxiredoxin redox state.

One possible explanation for the absence of transcriptional control in the *Lingulodinium* clock is the potential difficulty in regulating transcription in the dinoflagellates. Indeed, these organisms do not contain detectable levels of histone proteins [28], their chromosomes are permanently condensed [29], and the number and diversity of transcription factors is also much reduced compared to other eukaryotes [16,30]. In addition, a global analysis of mRNAs in the dinoflagellate *Karenia* has shown their half-lives to be substantially longer than reported in other organisms [31], as might be expected if little control was exerted at the level of RNA synthesis rates. *Lingulodinium* also appears to have quite stable RNAs, as least for the few specific cases that have been examined [32].

How might timing signals be generated in *Lingulodinium* in the absence of rhythmic transcripts? Previous work with the system has shown that inhibitors of translation (such as anisomycin, puromycin and cycloheximide) have major phase-shifting effects on the bioluminescence rhythm [33-35]. In particular, a specific dose of the inhibitor anisomycin given at a specific time, but not lower or higher doses, can induce arrhythmicity [35]. This has been interpreted by a limit cycle model to mean that the clock has been driven to a singularity and provides strong presumptive evidence that translation is a state variable in the clock mechanism. In addition to inhibitors of translation, the kinase inhibitors staurosporine and 6-dimethyl amino purine (6-DMAP) affect the period of the bioluminescence rhythm when administered chronically and strongly affect light-induced phase shifts [36]. Similarly, a range of protein phosphatase inhibitors (okadaic acid, calyculin A and cantharidin) also affect the rhythmic period when given chronically, although they appear unable to block light-induced phase shifts [37]. We suggest that, in the absence of rhythmic transcripts, *Lingulodinium* may have evolved translational/posttranslational feedback loops with 24-hour rhythmicity that can act as the central timing mechanism. One intriguing possibility is that protein phosphorylation may be used to control translation, as *Lingulodinium* has casein kinase 2 (CK2) sites in a large number of RNA binding proteins [38]. CK2 is a kinase that has been implicated in animal, plant and fungal clock mechanisms [39].

There is growing awareness that RNA-binding proteins may play an important role in clock function. For example, the *Chlamydomonas* protein CHLAMY1 is involved in controlling both the period and phase of the circadian rhythms [40]. In addition, a cold-induced RNA-binding protein appears to be required for efficient translation of the clock gene *Clock* transcripts in mammalian cells [41]. Lastly, ATX (ataxin-2) in *Drosophila* is an RNA-associated protein that, together with TYF (Twenty-four), is required for translating the transcripts from the clock gene *Period* (*Per*) [42,43]. TYF binds both to the poly(A)-binding protein (PABP), a factor binding the polyadenylated tail at the 3’ end of mRNA, and to the eukaryotic translation initiation factor eIF4E, which binds the cap at the 5’ end of the mRNA [43]. This circularization is thought to increase translation [44] which in this case would be targeted to the *Per* transcript. In mouse liver cells, changes in the length of the poly(A) tail occur independently from the steady state level of the transcripts, and tail length is associated with circadian control over protein synthesis [45].

The lack of rhythmic transcripts over the daily cycle in *Lingulodinium* provides new impetus for an examination of translation and post-translational mechanisms in the clock. The former may be addressed by methods such as translating ribosome affinity purification, which has been used to demonstrate rhythmic translation from constant levels of mRNA in drosophila [46]. The latter, at least for modifications involving phosphorylation, is now becoming accessible through phosphoprotein purification and tandem mass spectrometry (MS/MS) *de novo* sequencing [38]. In any event, it is clear that the long-studied *Lingulodinium* circadian system has still some surprises in reserve.

## Conclusions

The lack of oscillating RNAs over the circadian cycle of the dinoflagellate *Lingulodinium* indicates that the clock mechanism in this organism does not use or require rhythmic changes in RNA amounts.

## Methods

### Cell culture

Unialgal but not axenic *Lingulodinium polyedrum* (CCMP 1936, previously *Gonyaulax polyedra*) was obtained from the Provasoli-Guillard National Center for Marine Algae and Microbiota (East Boothbay, ME, USA). Cell cultures were grown in normal f/2 medium prepared using Instant Ocean under a 12-hour light (40 μmol photons m^−2^ s^−1^ cool white fluorescent light) and 12-hour dark regime at a temperature of 18 ± 1°C. This light/dark regime is termed LD, with LD 0 corresponding to lights on and LD 12 to lights off. Cells were harvested by filtration on Whatman 541 paper and washed with 200 ml of sterile seawater to reduce bacterial contamination.

### RNA extraction and RNA-Seq

RNA-Seq was performed for two different experiments. In one experiment, *Lingulodinium polyedrum* cells grown in f/2 seawater medium at 18°C under a 12:12 light:dark cycle [9] were isolated by centrifugation (500 g for one minute) at two times (ZT 6, ZT 18) and two times under constant light (CT 6, CT 18). The onset of the light phase in a light dark cycle or under continuous light is termed ZT 0 or CT 0, respectively. In another experiment, cells were taken at ZT 2, ZT 6, ZT 14 and ZT 18. All cell pellets were washed twice with fresh seawater and recentrifuged, and the cell pellets were resuspended in 1 mL of Trizol reagent (Life Technologies, Grand Island, NY, USA) and total RNA was extracted as per the manufacturer’s protocol. For the first experiment, RNA quality was assessed by Northern blots before and after purification of poly(A) RNA using the poly(A) tract mRNA isolation kit (Promega, Madison, WI, USA) and sequenced using a Genome Analyzer IIX (Illumina) at the McGill University and Génome Québec Innovation Center. For the second experiment, RNA pellets were dissolved in 50 μL diethylpyrocarbonate (DEPC) treated H_2_O, and a Bioanalyzer (Agilent, Santa Clara, CA, USA) test performed to assess the quality of the extracted RNA samples. An mRNA-Seq Sample Preparation Kit (Illumina) was used prior to sequencing of the mRNAs using a HiSeq platform (Illumina) at the Institut de Recherche en Immunologie et Cancérologie (Université de Montréal).

Reads were mapped to both a Velvet assembly (accession numbers JO692619 to JO767447) [16] and to a Trinity assembly (accession numbers GABP01000001 to GABP01114492) [17] using BWA (Burrows-Wheeler transformation) [47]. Sequencing produced 252 million 76 bp paired end reads (Illumina GAII, accession numbers SRR330443 to -6), of which 49.8% and 51.8% mapped to the Velvet and Trinity assemblies, respectively, and 545 million 100 bp paired end reads (HiSeq, accession numbers SRR1184543, 1184608, 1184657, 1184666) of which 95.5 and 88.3% mapped to the Velvet and Trinity assemblies, respectively. Read counts were analyzed by DESeq [19] and EdgeR [20] to uncover statistically significant differences**.** All reads and assemblies are available as a part of the National Center for Biotechnology Information BioProject PRJNA69549.

Northern blots were performed using total RNA isolated by Trizol extraction as described by the manufacturer. From the 131 sequences with DESeq significant differences, 67 pairs of oligonucleotides were designed from the Trinity assembly sequences and tested by PCR, with 27 pairs producing sequence-validated amplicons. The majority of these were from the same group (maximal levels at ZT 14) so only 17 were tested by Northern blotting, and of these 9 (including a mitochondrial *cox1* probe) yielded a well-defined signal after exposure. An additional two probes were prepared from previously cloned plastid-encoded sequences *atpA* and *psbC* [22]. An actin probe was used to control for RNA loading.

### Transcription inhibition

All the inhibitors were dissolved in 100% dimethyl sulfoxide (DMSO) to prepare the stock solution. To inhibit transcription, 20 μL from a stock of 10 mg/mL cordycepin (final concentration 20 μg/mL) and 5 μL from a stock of 1 mM actinomycin D (final concentration 0.5 μM) were added to a 10 mL culture of *Lingulodinium* cells. To monitor the inhibition of RNA synthesis, 5 μCi of ^32^P radionuclide was added after eight hours incubation with inhibitors. Total RNA was extracted using Trizol reagent (Life Technologies) after eight hours of incubation with the label. RNA pellets were dissolved in 50 μL of DEPC treated water and 2 μL aliquots mixed with 2 mL of scintillation fluid and counted using a scintillation counter (Perkin Elmer TriCarb 2800TR). The control was prepared by adding only the vehicle (DMSO) to the culture.

### Rhythm measurements

To monitor the bioluminescence rhythm after inhibition of transcription, cordycepin and actinomycin D were added at the same concentrations as above. After four hours of incubation of the cultures with the inhibitors, six 280 μL aliquots of cell culture were loaded into a 96-well microtiter plate. Control cells in seawater or with the vehicle alone (DMSO) were also included in the plate. The bioluminescence rhythm was monitored in constant darkness as described [17] using a Spectramax M5 (Molecular Devices, Sunnyvale, CA, USA) microplate reader kept in the culture room at constant temperature. Bioluminescence output was recorded for one second every two minutes for the next 72 hours (Additional file 3). Wells containing cultures were surrounded by wells filled with only seawater to limit evaporation. The pH rhythm was measured as described [48,49] using a pH electrode in a culture flask kept under constant light and temperature in the culture room. The treatments with DMSO alone or DMSO containing the inhibitors were repeated three times.

## Additional files

Additional file 1: Figure S1. Analysis using the Velvet assembly. **(A)** Transcript abundance for midday and midnight samples (as reads per kilobase per million, RPKM) obtained by mapping raw read data from the 100 bp read experiment to a 74,655 contig Velvet assembly. The fold difference for the contigs that showed a significant difference is shown as an inset (upper left) while the MA plot used to determine significant differences is at lower right. Contigs corresponding to rRNA have been removed. **(B)** Transcript abundance for ZT 18 and CT 18 analyzed as above for the 76 bp read experiment. Additional file 2: Figure S2. Analysis using duplicate ZT6 and ZT/CT18 samples. **(A)** Time of sampling in a light/dark cycle and constant light. **(B)** MA plots using ZT 6/CT 6 samples and ZT 18/CT 18 pairs as duplicates were analyzed by DESeq. There are no significant differences (*P*_adj_ <0.001) when mapped to the Trinity assembly. (C) Three significant differences are identified when mapped to the Velvet assembly. Additional file 3: Figure S3. Individual bioluminescence traces. The three individual bioluminescence traces used to determine the average of inhibitor-treated cultures shown in Figure S3B.
