# Supplementary figures and images for: The Lingulodinium circadian system lacks rhythmic changes in transcript abundance
Source: BMC Biol. 2014 Dec 20;12:107. doi: 10.1186/s12915-014-0107-z (PMC4298066; doi:10.1186/s12915-014-0107-z)

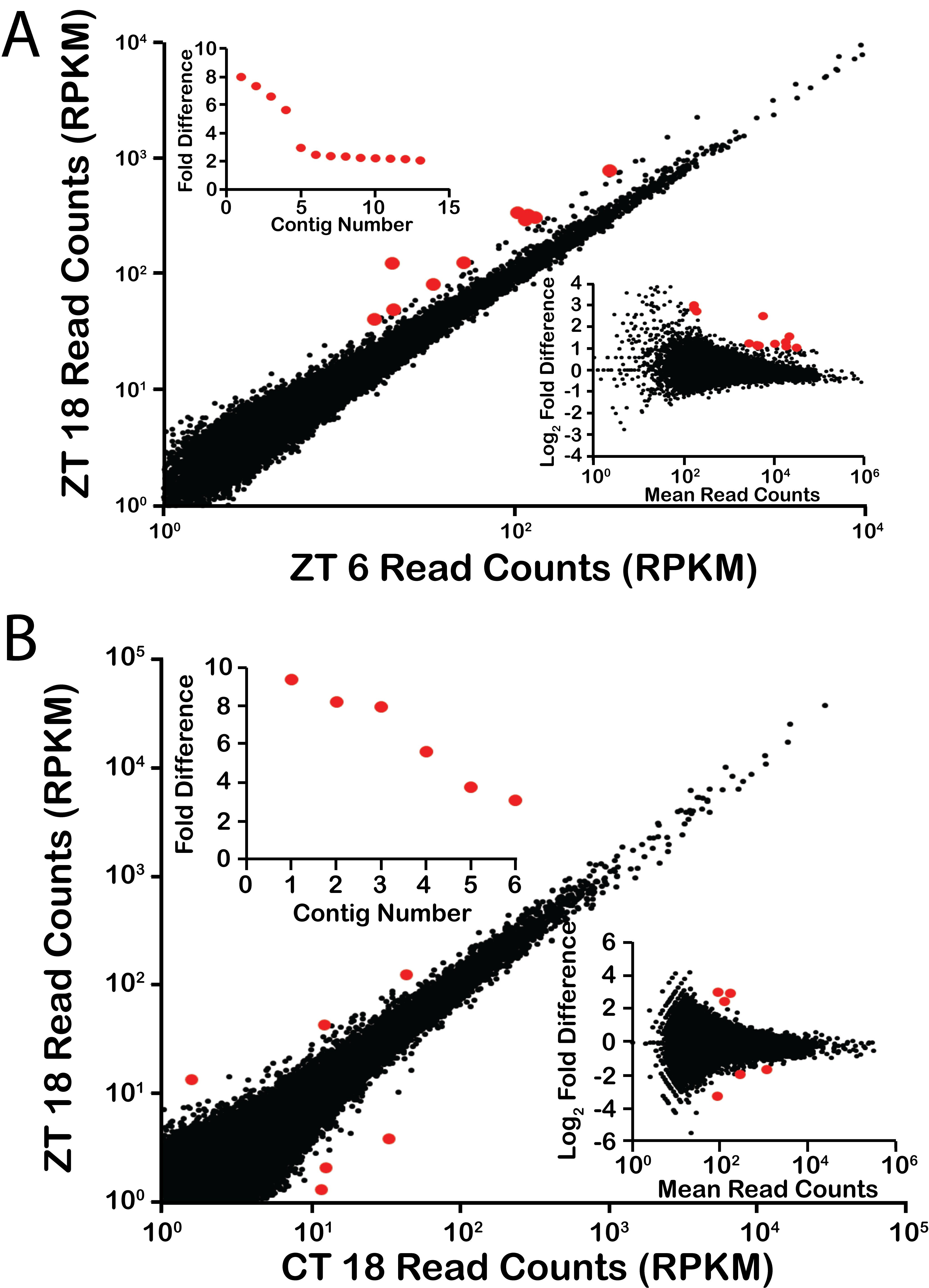

Supplement: Additional file 1: Figure S1. — Analysis using the Velvet assembly. (A) Transcript abundance for midday and midnight samples (as reads per kilobase per million, RPKM) obtained by mapping raw read data from the 100 bp read experiment to a 74,655 contig Velvet assembly. The fold difference for the contigs that showed a significant difference is shown as an inset (upper left) while the MA plot used to determine significant differences is at lower right. Contigs corresponding to rRNA have been removed. (B) Transcript abundance for ZT 18 and CT 18 analyzed as above for the 76 bp read experiment. [file 12915_2014_107_MOESM1_ESM.tiff]

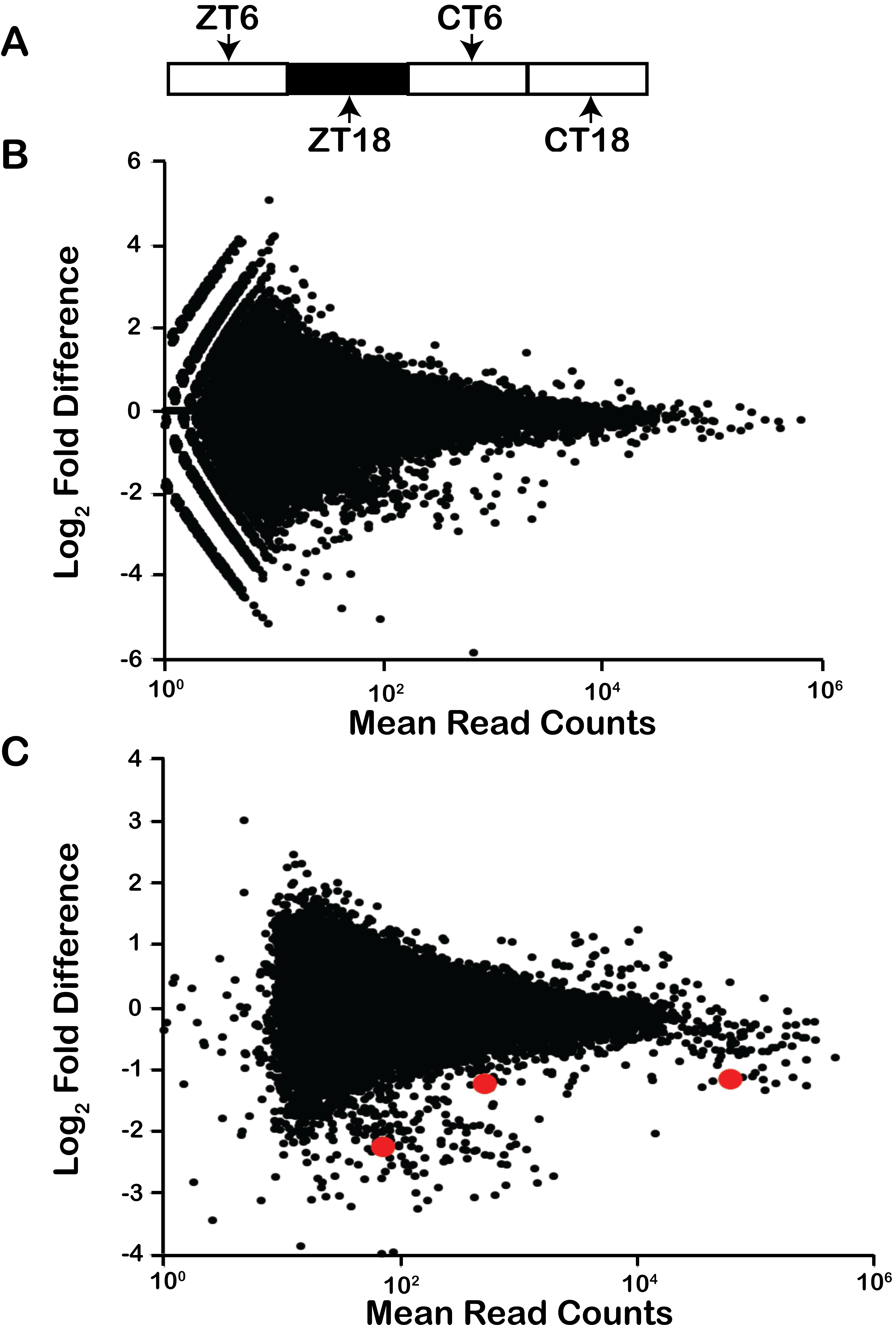

Supplement: Additional file 2: Figure S2. — Analysis using duplicate ZT6 and ZT/CT18 samples. (A) Time of sampling in a light/dark cycle and constant light. (B) MA plots using ZT 6/CT 6 samples and ZT 18/CT 18 pairs as duplicates were analyzed by DESeq. There are no significant differences (P adj <0.001) when mapped to the Trinity assembly. (C) Three significant differences are identified when mapped to the Velvet assembly. [file 12915_2014_107_MOESM2_ESM.tiff]

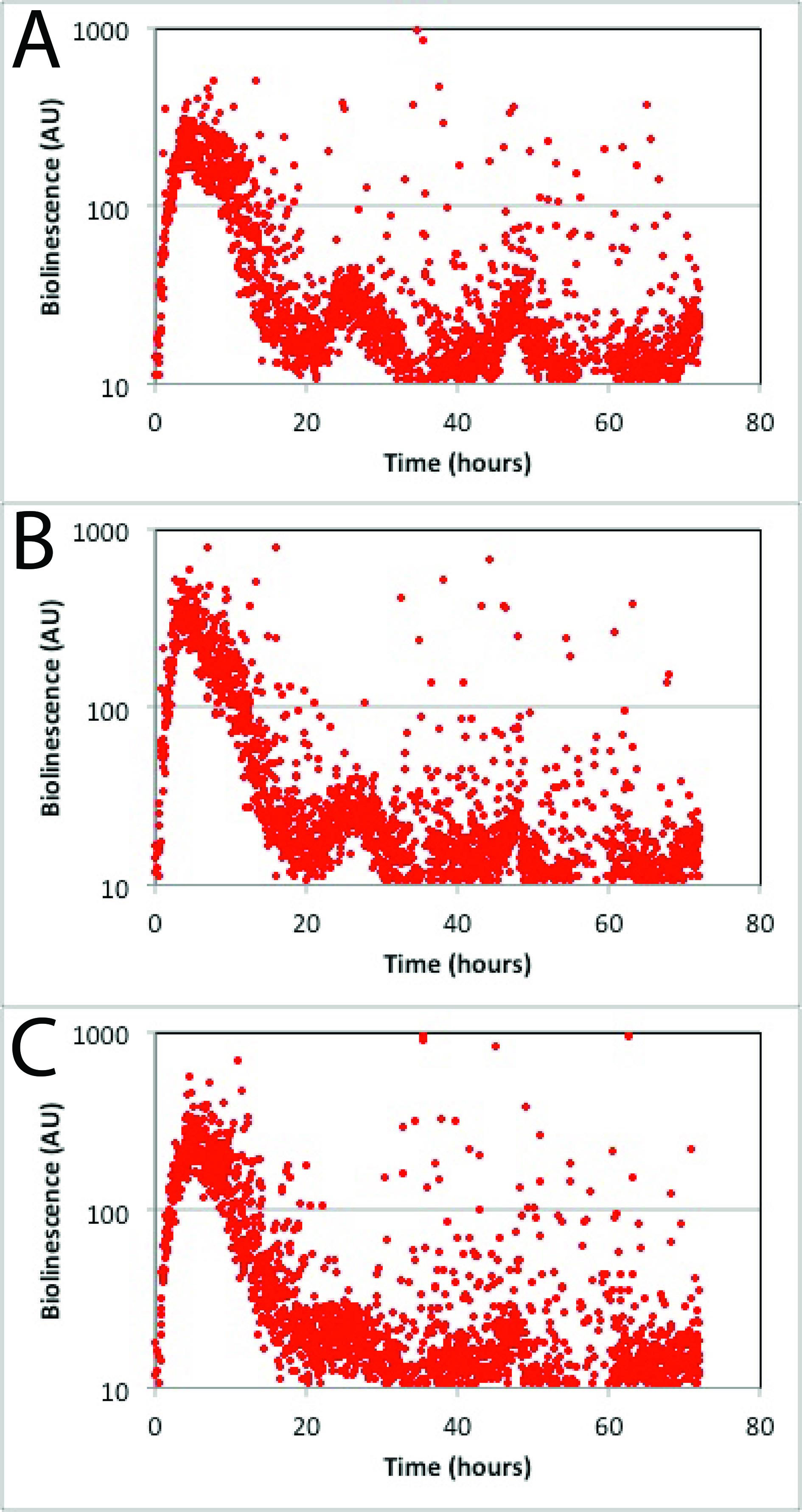

Supplement: Additional file 3: Figure S3. — Individual bioluminescence traces. The three individual bioluminescence traces used to determine the average of inhibitor-treated cultures shown in Figure S3B. [file 12915_2014_107_MOESM3_ESM.jpeg]
